# MCM8 promotes gastric cancer progression through RPS15A and predicts poor prognosis

**DOI:** 10.1002/cam4.7424

**Published:** 2024-07-10

**Authors:** Lixian Ding, Mingjun Sun, Yanyan Sun, Jinxing Li, Zhicheng Zhang, Shuwei Dang, Jinning Zhang, Bang Yang, Youlin Dai, Qinghao Zhou, Dazhi Zhou, Encheng Li, Shuqi Peng, Guodong Li

**Affiliations:** ^1^ Department of General Surgery The Fourth Affiliated Hospital of Harbin Medical University Harbin People's Republic of China; ^2^ Bio‐Bank of Department of General Surgery The Fourth Affiliated Hospital of Harbin Medical University Harbin People's Republic of China; ^3^ Surgery Teaching and Research Office The Fourth Affiliated Hospital of Harbin Medical University Harbin People's Republic of China; ^4^ Department of General Surgery The Second Affiliated Hospital of Harbin Medical University Harbin People's Republic of China

**Keywords:** gastric cancer, MCM8, prognosis, proliferation, RPS15A

## Abstract

**Background:**

Gastric cancer (GC) is the fourth leading cause of cancer‐related death worldwide. Minichromsome maintenance proteins family member 8 (MCM8) assists DNA repair and DNA replication. MCM8 exerts tumor promotor function in multiple digestive system tumors. MCM8 is also considered as a potential cancer therapeutic target.

**Methods:**

Bioinformatics methods were used to analyze MCM8 expression and clinicopathological significance. MCM8 expression was detected by immunohistochemistry (IHC) staining and qRT‐PCR. MCM8 functions in GC cell were explored by Celigo cell counting, colony formation, wound‐healing, transwell, and annexin V‐APC staining assays. The target of MCM8 was determined by human gene expression profile microarray. Human phospho‐kinase array kit evaluated changes in key proteins after ribosomal protein S15A (RPS15A) knockdown. MCM8 functions were reassessed in xenograft mouse model. IHC detected related proteins expression in mouse tumor sections.

**Results:**

MCM8 was significantly upregulated and predicted poor prognosis in GC. High expression of MCM8 was positively correlated with lymph node positive (*p* < 0.001), grade (*p* < 0.05), AJCC Stage (*p* < 0.001), pathologic T (*p* < 0.01), and pathologic N (*p* < 0.001). MCM8 knockdown inhibited proliferation, migration, and invasion while promoting apoptosis. RPS15A expression decreased significantly after MCM8 knockdown. It was also the only candidate target, which ranked among the top 10 downregulated differentially expressed genes (DEGs) in sh‐MCM8 group. RPS15A was identified as the target of MCM8 in GC. MCM8/RPS15A promoted phosphorylation of P38α, LYN, and p70S6K. Moreover, MCM8 knockdown inhibited tumor growth, RPS15A expression, and phosphorylation of P38α, LYN, and p70S6K in vivo.

**Conclusions:**

MCM8 is an oncogene and predicts poor prognosis in GC. MCM8/RPS15A facilitates GC progression.

## Graphical abstract

MCM8 is an oncogene and predicts poor prognosis in gastric cancer (GC) patients. The tumor promotor function of MCM8 in gastric cancer is realized by binding with RPS15A. MCM8/RPS15A axis facilitated phosphorylation of many kinases in GC, including P38α, LYN, and p70S6K. This is the first study to determine the function of MCM8/RPS15A axis through proliferation, migration, and apoptosis in GC.

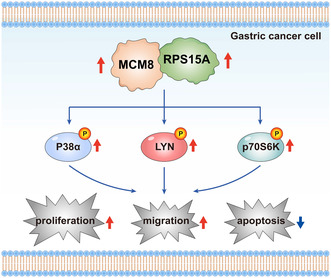

## 1 INTRODUCTION

Gastric cancer (GC) is a common cancer, which ranks fifth in the morbidity and fourth in the mortality of cancer worldwide.^1^ The factors of GC initiation contain inevitable hazard factors like age and controllable factors like helicobacter pylori infection.^2^, ^3^ Surgical resection is the best therapeutic method for GC currently.^4^ However, most GC patients have lost the optimal time for surgery.^5^ Adjuvant therapies especially targeted therapy are effective for advanced GC.^6^, ^7^ Nevertheless, the selection and indications of targeted drugs were limited for GC.^8^, ^9^ Therefore, the mechanisms of GC progression are required to explore to promote the development of targeted therapy.

Minichromsome maintenance proteins (MCMs) have a similar structure with a domain of 200 amino acid residues called MCM box.^10^ MCMs promote the initiation and elongation of DNA replication in eukaryotes.^11^ They play the role of helicase and assist RAD51 recombinase to promote the homologous recombination (HR).^12^ Recent studies indicated that MCMs affect the progression of digestive system tumors.^13^, ^14^, ^15^ MCM5 and MCM7 inhibit DNA replication and tumorigenesis in hepatocellular carcinoma (HCC).^13^ MCM7 binds with PRMT5 to promote phenotypic functions of colorectal cancer (CRC).^14^ MCM6 promotes HCC metastasis via MEK/ERK pathway.^15^ These studies showed that MCMs are involved in the progression of digestive system tumors. Therefore, it is necessary to further study the functions of MCMs in other gastrointestinal cancers.

MCM8 is a member of MCMs. It is crucial in cell cycle, DNA repair, and DNA replication.^16^, ^17^ Compared to other members of the MCM family, MCM8 has been shown to promote cancer progression, such as bladder cancer and CRC.^18^, ^19^ Additionally, numerous studies have demonstrated that MCM8 facilitates progression of various cancers.^20^, ^21^ Previous studies have only confirmed that MCM8 promotes the phenotypic function of GC cell. The mechanisms of MCM8 in GC are still unknown.^22^ In this study, MCM8 was significantly increased in GC. High MCM8 expression was positively correlated with poor prognosis. MCM8 knockdown inhibited the proliferation, migration, and invasion while promoting GC cell apoptosis. We further found that MCM8 promoted GC process through ribosomal protein S15A (RPS15A). Moreover, MCM8/RPS15A axis facilitated phosphorylation of P38α, LYN, and p70S6K. Collectively, MCM8 is an oncogene and promotes GC through RPS15A.

## 2 MATERIALS AND METHODS

### 2.1 Human samples

A total of 180 GC and normal gastric tissues were obtained from the Fourth Affiliated Hospital of Harbin Medical University. The informed consent was achieved from patients. All study methodologies were strictly in accordance with the Helsinki Declaration for the Use of Human Subjects and approved by the Ethics Committee at the Fourth Affiliated Hospital of Harbin Medical University (YXLLSC‐2018‐01). We obtained paired tissues from 81 GC patients and 18 GC tissues. There were 15 cases with detachment tissues and 18 cases with incomplete pathological data. Thus, 147 tissues were used for statistical analysis.

### 2.2 Data analysis of the TCGA database

RNA‐seq counts and clinical information of samples were downloaded from The Cancer Genome Atlas (TCGA) and GTEx. After data screening, 408 GC and 211 normal samples were used for data analysis.

### 2.3 Cell culture

GC cell lines (AGS, MGC‐803, MKN‐45, HGC‐27, and SGC‐7901) and gastric mucosa epithelial cell line GES‐1 were bought from Shanghai Institutes for Biological sciences, China. AGS was cultured in F‐12K medium containing 10% fetal bovine serum (FBS) (ThermoFisher Scientific, USA) and 1% penicillin–streptomycin (P–S) (HyClone). HGC‐27 was maintained in RPMI‐1640 medium with 20% FBS and 1% P–S. MGC‐803, MKN‐45, and SGC‐7901 were cultured in RPMI‐1640 medium with 10% FBS and 1% P–S, respectively. All cells were kept in incubator with 37°C and 5% CO_2_. The expression level of MCM8 was higher in MGC‐803, AGS, and SGC‐7901 cells.

### 2.4 Cell transfection

Lentiviral vector carrying sh‐MCM8 RNA or sh‐RPS15A RNA were constructed. AGS and MGC‐803 cells were transfected with GFP fluorescent labeled lentiviral vectors. The fluorescence expression was observed by fluorescence microscope to evaluate the efficiency of cell infection. The expression of MCM8 and RPS15A after transfection was detected by qRT‐PCR and WB.

### 2.5 Quantitative real time PCR (qRT‐PCR)

Total RNA was extracted from cells using TRIzol reagent (Invitrogen, Waltham, MA, USA). Reverse transcription was performed using M‐MLV Reagent Kit (Promega Corporation, Madison, USA). qRT‐PCR reactions were implemented with SYBR Green Master Mix kit (Takara, Otsu, Japan). The 2^−ΔΔCt^ method was used for relative quantification and statistical analysis. The primers for gene application: MCM8, 5'‐ATGGCTTTTCTTTGTGCTGC‐3′ (F), 5'‐CCAGTCCATCGTAACTGTGAGA‐3′ (R); RPS15A, 5'‐CGCGCCGCCACAATG‐3′ (F), 5'‐CACAGTGAGAAACCGGACGA‐3′ (R); CDK4, 5'‐CTACCAGATGGCACTTACACCC‐3′ (F), 5'‐GCAAAGATACAGCCAACACTCC‐3′ (R); c‐Jun, 5'‐TGCCTCCAAGTGCCGAAAA‐3′ (F), 5'‐TAAGCTGTGCCACCTGTTCC‐3′ (R); MAPK14, 5'‐GCCTACTTTGCTCAGTACCACG‐3′ (F), 5'‐TCATCATAGGTCAGGCTTTTCC‐3′ (R); CCND1, 5'‐AGGCGGAGGAGAACAAACAGA‐3′ (F), 5'‐GGAGGGCGGATTGGAAATGAA‐3′ (R); SMAD3, 5'‐ATGTCGTCCATCCTGCCTTTC‐3′ (F), 5'‐CCTTCTCGCACCATTTCTCCT‐3′ (R); SMAD4, 5'‐GACCACGCGGTCTTTGTACA‐3′ (F), 5'‐CGATGACACTGACGCAAATCAA‐3′ (R); ARAF, 5'‐CGGTAGTAGAGGAGGTAGTGATGG‐3′ (F), 5'‐TGCTGGTGACTTGGAATGTG‐3′ (R); ORC1, 5’‐AAAAGCCCAGAATGAAGC‐3′ (F), 5'‐TTACCTAGAAACCGAAGC‐3′ (R); GNB1, 5'‐GCTGTTTGACCTTCGTGCTG‐3′ (F), 5'‐CAGTTGAAGTCGTCGTACCCA‐3′ (R); PPP1CB, 5'‐TTGTGCAGATGACTGAAGCAGAAGTT‐3′ (F), 5'‐CCAAAAGAATAGGCTGGCTGAGAAA‐3′ (R); RPS6KA1, 5'‐CTGAAGAAGGCAACGCTGAAAGTA‐3′ (F), 5'‐ACGCAGGAAGTCCAGAATGAGAT‐3′ (R); RPS6KB2, 5'‐CCTGGCTGAGATCACGCTG‐3′ (F), 5'‐AGAGTCCAAAGTCGGTCAGTTT‐3′ (R); LYN, 5'‐AGAGCGATGAAGGTGGCAAAG‐3′ (F), 5'‐GACTCGGAGACCAGAACATTAGC‐3′ (R); GAPDH, 5'‐TGACTTCAACAGCGACACCCA‐3′ (F), 5'‐CACCCTGTTGCTGTAGCCAAA‐3′ (R).

### 2.6 Western blot (WB)

Total proteins were extracted with RIPA. The target proteins were separated by SDS‐PAGE and transferred to PVDF membrane. The PVDF membrane was dipped in the blocking solution (TBST solution containing 5% skim milk) at room temperature for 2 h. The blots were then incubated with various primary antibodies at 4°C overnight. TBST solution was used to wash the PVDF membrane for three times. The membrane was incubated with the corresponding secondary antibody at room temperature for 2 h. Next, the membrane was washed with TBST solution for three times. Protein bands were visualized by ECL reagent (Bio‐Rad, Hercules, Canada) and imaged with the ChemiDoc XRS System (Bio‐Rad, Hercules, CA, USA). Image J was used to analyze the proteins. The antibodies are listed below. MCM8 (1:500, Proteintech, Chicago, USA), RPS15A (1:2000, Proteintech, Chicago, USA), CDK4 (1:1000, Proteintech, Chicago, USA), MAPK14 (1:2000, Proteintech, Chicago, USA), CCND1 (1:750, CST, Boston, USA), SMAD3 (1:1000, Abcam, Cambridge, UK), SMAD4 (1:500, Santa Cruz, California, USA), ARAF (1:1000, CST, Boston, USA), RPS6KA1 (1:500, Proteintech, Chicago, USA), GAPDH (1:30000, Proteintech, Chicago, USA), P53 (1:3000, Proteintech, Chicago, USA), p‐P53 (1:2000, Proteintech, Chicago, USA), STAT3 (1:1500, CST, Boston, USA), p‐STAT3 (1:500, CST, Boston, USA), c‐Jun (1:1000, Proteintech, Chicago, USA), p‐c‐Jun (1:2000, CST, Boston, USA), p70S6K (1:1000, Affinity, Cincinnati, USA), p‐p70S6K (1:1000, Affinity, Cincinnati, USA), P38α(1:2000, Abcam, Cambridge, UK), p‐P38α(1:1000, Abcam, Cambridge, UK), LYN (1:1000, CST, Boston, USA), p‐LYN (1:1000, CST, Boston, USA), PYK2 (1:2000, Abcam, Cambridge, UK), p‐PYK2 (1:1000, CST, Boston, USA), STAT1 (1:1000, Proteintech, Chicago, USA), p‐STAT1 (1:2000, Abcam, Cambridge, UK).

### 2.7 Celigo cell counting assay

Cells were cultured in an incubator with a density of 2000 cells per well. The cell images were taken by Celigo image cytometer (Nexcelom Bioscience, Lawrence, USA) 24 h after culturing. The plates were examined everyday continuously for 5 days. The number of cells with green fluorescence in each scanning well plate was calculated. The 5‐day cell proliferation curve was drawn.

### 2.8 Colony formation assay

GC cells were plated in six‐well culture plates at 1000 cells/well. The cells were incubated at 37°C for 2 weeks. Then, the cells were washed two times with phosphate buffered saline (PBS) solution and stained with Giemsa (Shanghai Dingguo, Shanghai, China). The number of colonies containing ≥50 cells was counted under a microscope (Olympus, Japan).

### 2.9 Cell apoptosis assay

Cells were inoculated in six‐well plate and cultured continuously for 5 days. The cells were centrifuged at 1500 rpm for 5 min. Then, they were washed with D‐Hanks and 1 × binding buffer (eBioscience, California, USA), respectively. The cells precipitation were resuspended in 200 μL of 1 × binding buffer and stained with 5 μL of Annexin V (eBioscience, California, USA) in the dark for 15 min. The apoptosis cells were tested by flow cytometry (Millipore, Massachusetts, USA).

### 2.10 Cell cycle assay

Changes in cell cycle distribution were determined by fluorescence‐activated cell sorting. GC cells were inoculated in six‐well plate and cultured continuously for 5 days. They were centrifuged at 1200 rpm for 5 min and washed with PBS. Then, the cells were fixed in ice‐cold 70% ethanol for 30 min. Following fixation, cells were resuspended in 1 × PBS, treated with 100 × RNase (10 mg/mL), and stained with 40 × propidium iodide (2 mg/mL) for 30 min. The cell cycle was detected by flow cytometry (Millipore, Massachusetts, USA).

### 2.11 Wound‐healing assay

The successfully transfected cells were added to the six‐well plate and allowed to grow to confluence. Scratches were scraped upward from the center with scratch tester and cultured with low concentration serum medium. The cell mobility of each group was calculated after 24 h. The migration rate was calculated and analyzed under fluorescence‐based Cellomics ArrayScan VTI analyzer (Thermo Fisher Scientific, Massachusetts, USA).

### 2.12 Transwell assay

Roughly 1.0 × 10^5^ cells from serum‐free medium were placed in transwell chambers and transferred to the lower chambers of medium containing 10% FBS. The cells without metastasis were removed after 24 h. The transferred cells attached to the lower surface of the membrane insert were stained using Giemsa (Shanghai Dingguo, Shanghai, China) and quantified. The invasive capacity of GC cells was confirmed by transwell chambers with 100 μL of matrige.

### 2.13 Co‐immunoprecipitation assay (Co‐IP)

Cells are cleaved and proteins are extracted. The lysate of 1 mg total protein was rotated and incubated with the appropriate amount of antibody at 4°C overnight. Then, 20 μL beads were placed into a 1.5 mL EP tube containing 1 mL PBS. The supernatant was discarded. The above post‐incubation protein lysates were added and rotated for incubation at 4°C for 1 h. The precipitated samples were washed three times and were further analyzed by WB using the indicated antibodies.

### 2.14 Animal assay

Four‐week‐old BABL/c female nude mice were purchased from Shanghai Lingchang Biological Technology, China and randomly divided into different groups (sh‐MCM8 and sh‐Ctrl, *n* = 10 per group). MGC‐803 cells suspension was prepared with the concentration of 1 × 10^7^ cells/mL. Then, 500 ul MGC‐803 cell suspension containing 5 × 10^6^ cells was injected into the right forelimb axillary in nude mice to construct subcutaneous GC model. Tumor volume was calculated according to the following formula. Tumor volume (mm^3^) = π/6 × L × W × W (W, width at the widest point; L, perpendicular width). The expression of MCM8 and the size of the tumor were observed by in vivo imaging of small animals after 4 weeks. The weight of tumor and expression of MCM8 were detected after sacrifice. Tumor tissues were embedded, resected, and stained with HE and Ki‐67 to evaluate proliferation. Tumor tissues also were used to detect MCM8, RPS15A, P38α, p‐P38α, LYN, p‐LYN, p70S6K, and p‐p70S6K protein.

### 2.15 Statistical analysis

All statistical analyses were performed with GraphPad Prism 8.0.1 software. Overall survival (OS) curves were constructed using the Kaplan–Meier method and analyzed by the log‐rank test. The Mann–Whitney *U* and Spearman test were used to statistically analyze the correlation between MCM8 expression in cancer tissues and clinicopathological features. Experimental data were presented as the mean ± standard deviation. Statistical comparisons between two experimental groups were performed using student's *t*‐test. Statistical comparisons between two experimental groups were performed using student's *t*‐test. The qRT‐PCR data were analyzed by the 2^−ΔΔCT^ method. *p* values are represented as asterisks on graphs (*p < 0.05; **p < 0.01; ***p < 0.001). All experimental values represent a minimum of three individual experiments.

## 3 RESULTS

### 3.1 MCM8 is significantly upregulated and predicts poor prognosis in GC patients

**FIGURE 1:**
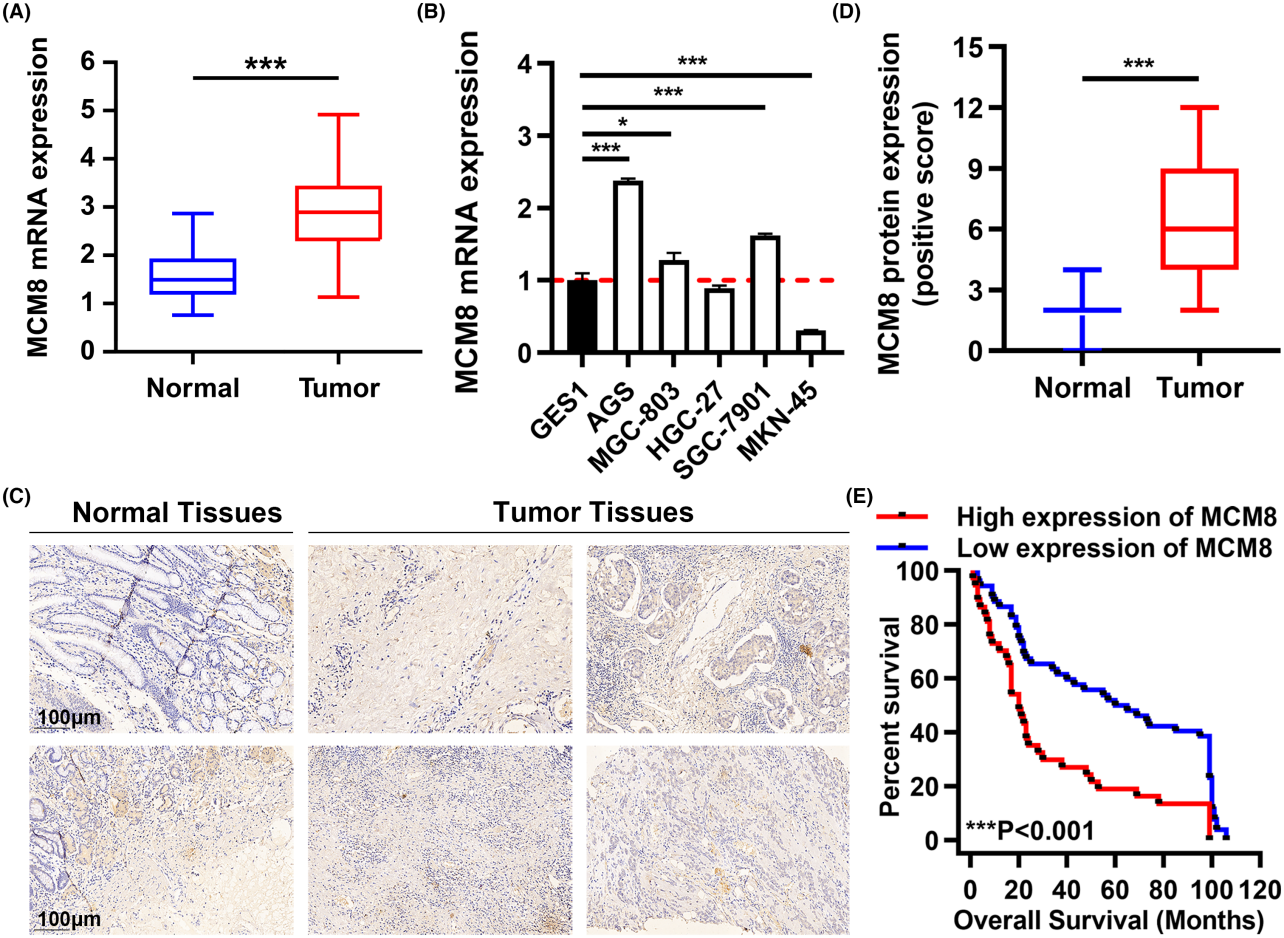
MCM8 is significantly elevated in GC and predicts poor prognosis. (A) MCM8 expression in GC (*n* = 408) and normal tissues (*n* = 211) from TCGA and GTEx. (B) MCM8 expression in GC and GES‐1 cells. (C, D) IHC staining of MCM8 in GC and normal tissues. Scale bar: 100 μm. (E) Kaplan–Meier plots of the overall survival (OS) rate of GC patients. *p < 0.05, **p < 0.01, and ***p < 0.001.

To evaluate the expression of MCM8 in GC, we first downloaded RNA‐seq counts of 408 GC and 211 normal samples from TCGA and GTEx. MCM8 expression was significantly increased in GC tissues (Figure 1A). The expression of MCM8 was increased in most GC cells (Figure 1B). Moreover, our clinical data confirmed the high expression of MCM8 in GC (Table 1). The IHC staining of MCM8 verified the high expression of MCM8 protein in GC tissues (Figure 1C,D). According to the Mann–Whitney *U* (Table 2) and Spearman correlation analysis (Table 3), MCM8 expression was positively correlated with T infiltrate, lymph node metastasis N, AJCC stage, and pathological grade. The Kaplan–Meier survival analysis showed that high expression of MCM8 was correlated with low OS rate in GC patients (Figure 1E). In conclusion, MCM8 is significantly elevated and predicts poor prognosis in GC.

**TABLE 1:** MCM8 expression in gastric cancer tissues and para‐carcinoma tissues.

MCM8 expression	Tumor tissue		Para‐carcinoma tissue		*p* value
	Cases	Percentage	Cases	Percentage	
Low	49	60.5	66	100	*p* < 0.01**
High	32	39.5	0	0	

**TABLE 2:** Relationship between MCM8 expression and clinicopathological features in patients with GC by Mann–Whitney *U* analysis.

Features	Cases	MCM8 expression		*p* Value
		Low	High	
All patients	81	49	32	
Age (years)				
≤65	42	29	13	0.104
>65	39	20	19	
Gender				
Male	52	35	17	0.095
Female	29	14	15	
Tumor metastasis				
No	74	47	27	0.072
Yes	7	2	5	
Lymph node positive				
≤4	44	36	8	*** *p* < 0.001
>4	37	13	24	
Tumor size				
≤5	41	27	14	0.321
>5	40	22	18	
Grade				
II	12	11	1	* *p* < 0.05
III	61	35	26	
IV	8	3	5	
AJCC stage				
1	7	7	0	*** *p* < 0.001
2	28	26	2	
3	40	14	26	
4	6	2	4	
Pathologic T				
T1	7	7	0	** *p* < 0.01
T2	5	4	1	
T3	53	32	21	
T4	16	6	10	
Pathologic N				
N0	23	21	2	*** *p* < 0.001
N1	12	9	3	
N2	21	13	8	
N3	25	6	19	
Ki67 expression				
Low	45	27	18	0.920
High	36	22	14	

* *p* < 0.05.

** *p* < 0.01.

*** *p* < 0.001.

**TABLE 3:** Relationship between MCM8 expression and clinicopathological features in patients with GC by Spearman correlation analysis.

Features	*p* value	Spearman correlation coefficient
Lymph node positive	*p* < 0.001***	0.476**
Grade	*p* < 0.05*	0.286**
AJCC stage	*p* < 0.001***	0.576**
T infiltrate	*p* < 0.01**	0.323**
Pathologic N	*p* < 0.001***	0.538**

### 3.2 MCM8 knockdown suppresses the proliferation, migration, invasion, and facilitates apoptosis of GC cells in vitro

**FIGURE 2:**
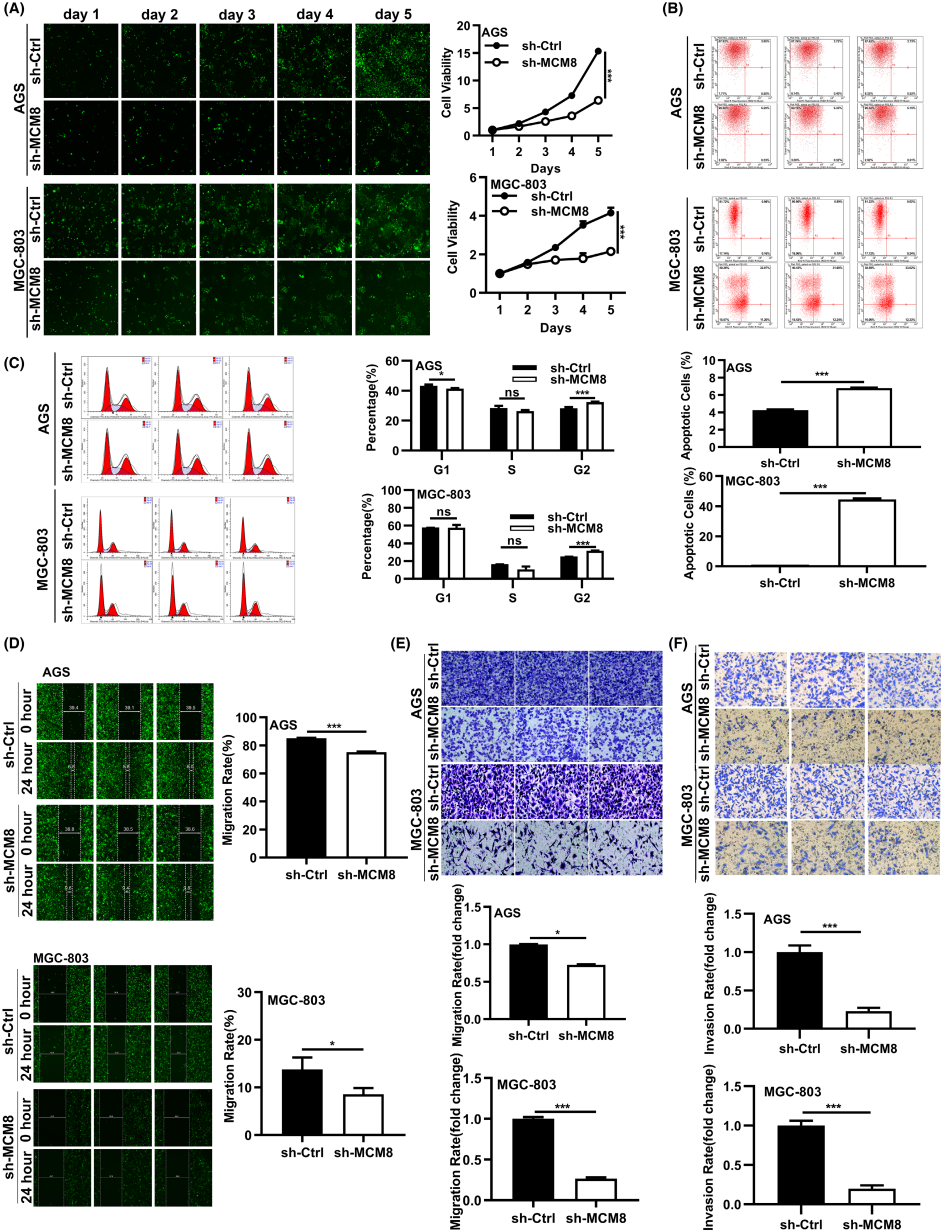
MCM8 knockdown suppresses the phenotypic functions of GC. (A) Celigo cell counting assay was used to analyze proliferation of MGC‐803 and AGS cells after MCM8 knockdown. (B, C) Flow cytometry was performed to detect (B) cell apoptosis and (C) cell cycle of MGC‐803 and AGS cells. (D–F) MGC‐803 and AGS cells migration and invasion ability was accessed by (D) Wound‐healing assay (scale bar: 1 μm) and (E, F) Transwell assay (200×). *p < 0.05, **p < 0.01, and ***p < 0.001.

We next investigated the functions of MCM8 in GC. We decreased MCM8 expression in MGC‐803 and AGS cells with short hairpin RNA (sh‐RNA). qRT‐PCR and WB presented the downregulation of MCM8 at mRNA and protein levels (Figure S1A,B). Celigo cell counting assay was performed to evaluate the proliferation of AGS and MGC‐803 cells after MCM8 knockdown. The results showed that the proliferation of GC cells decreased after MCM8 knockdown (Figure 2A). To elucidate the effect of MCM8 on apoptosis and cell cycle, we performed Annexin V staining and flow cytometry on above two cells. Flow cytometry results showed that the proportion of apoptotic cells in sh‐MCM8 group was increased sharply (Figure 2B). The proportion of cells in G2 phase was elevated after MCM8 knockdown (Figure 2C). Moreover, MCM8 knockdown decreased the migration and invasion capacity of GC cells (Figure 2D–F). Subsequent KEGG pathway enrichment analysis showed that MCM8 co‐expression genes were mostly enriched in cell cycle and DNA replication pathway (Figure S2A). Meanwhile, we overexpressed MCM8 in AGS cells and examined phenotypic functions. qRT‐PCR and WB showed that MCM8 was overexpressed in AGS cells (Figure S1C,D). The phenotypic functions of GC cells after MCM8 overexpression were detected (Figure S3A–E). Therefore, MCM8 facilitates the proliferation, migration, invasion, and suppresses apoptosis of GC cells in vitro.

### 3.3 RPS15A is the downstream target of MCM8 in GC

**FIGURE 3:**
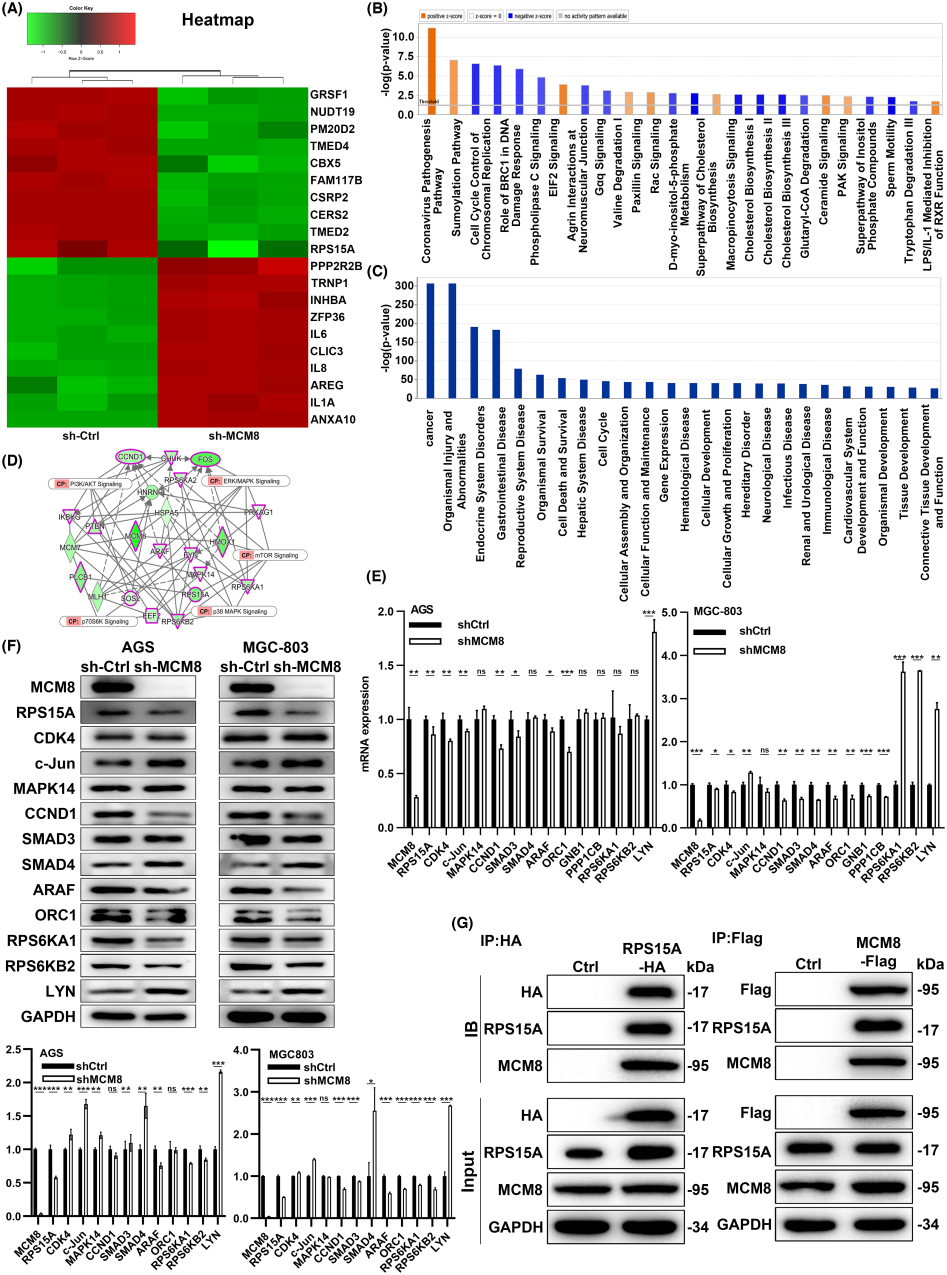
RPS15A is the target of MCM8 in GC. (A) The differentially expressed genes (DEGs) between sh‐MCM8 and sh‐Ctrl groups. (B) Ingenuity pathway analysis (IPA) analyzed the enrichment of DEGs in the typical signal pathways. (C) The significant enrichment of DEGs in diseases and functions. (D) Network of interactions between MCM8 and classic signaling pathway genes. The expression of targets in GC cells were detected by (E) qRT‐PCR and (F) WB. (G) The binding of MCM8 and RPS15A protein was confirmed via Co‐IP assay. *p < 0.05, **p < 0.01, and ***p < 0.001.

The potential mechanisms of MCM8 in GC were further studied. We performed human genome sequencing for sh‐MCM8 and sh‐Ctrl groups by Human Gene Expression Profile Microarray. Subsequently, we screened 838 differentially expressed genes (DEGs) between two groups with criterion of fold change ≥1.3 and false discovery rate <0.05. The 838 DEGs included 428 upregulated genes and 410 downregulated genes. Then, we made hierarchical clustering heat maps of two groups (Figure 3A). The heat maps showed the top 20 DEGs. Then, ingenuity pathway analysis (IPA) analyzed the enrichment of the DEGs in the typical signal pathways. It showed that cell cycle control of chromosomal replication, role of BRCA1 in DNA damage response, and phospholipase C signaling pathway were inhibited remarkably (Figure 3B). IPA also revealed that cancer was the most significantly enriched disease (Figure 3C). Moreover, we constructed a network of interactions between classic signaling pathway genes and MCM8 (Figure 3D). Next, qRT‐PCR was performed to detect the expression of key genes in these pathways after MCM8 knockdown (Figure 3E). The expression of candidate target was verified by WB (Figure 3F). RPS15A was expressed differentially in qRT‐PCR and WB. Meanwhile, RPS15A was the only candidate target, which ranked among the top 10 downregulated DEGs. Therefore, RPS15A was identified as the target of MCM8 in GC. Then, KEGG pathway enrichment analysis demonstrated that RPS15A co‐expression genes were mostly enriched in ribosomal function pathway (Figure S2B). It also performed for MCM8 and RPS15A co‐expression genes. Based on the common function of MCMs, we conducted Co‐IP assay on MCM8 and RPS15A. It confirmed the binding of RPS15A and MCM8 (Figure 3G). Collectively, RPS15A is the target of MCM8 in regulating GC. MCM8 may regulate RPS15A through transcriptional regulation and protein binding.

### 3.4 RPS15A knockdown inhibits the proliferation, migration, and invasion of GC cells while promoting GC cells apoptosis in vitro

**FIGURE 4:**
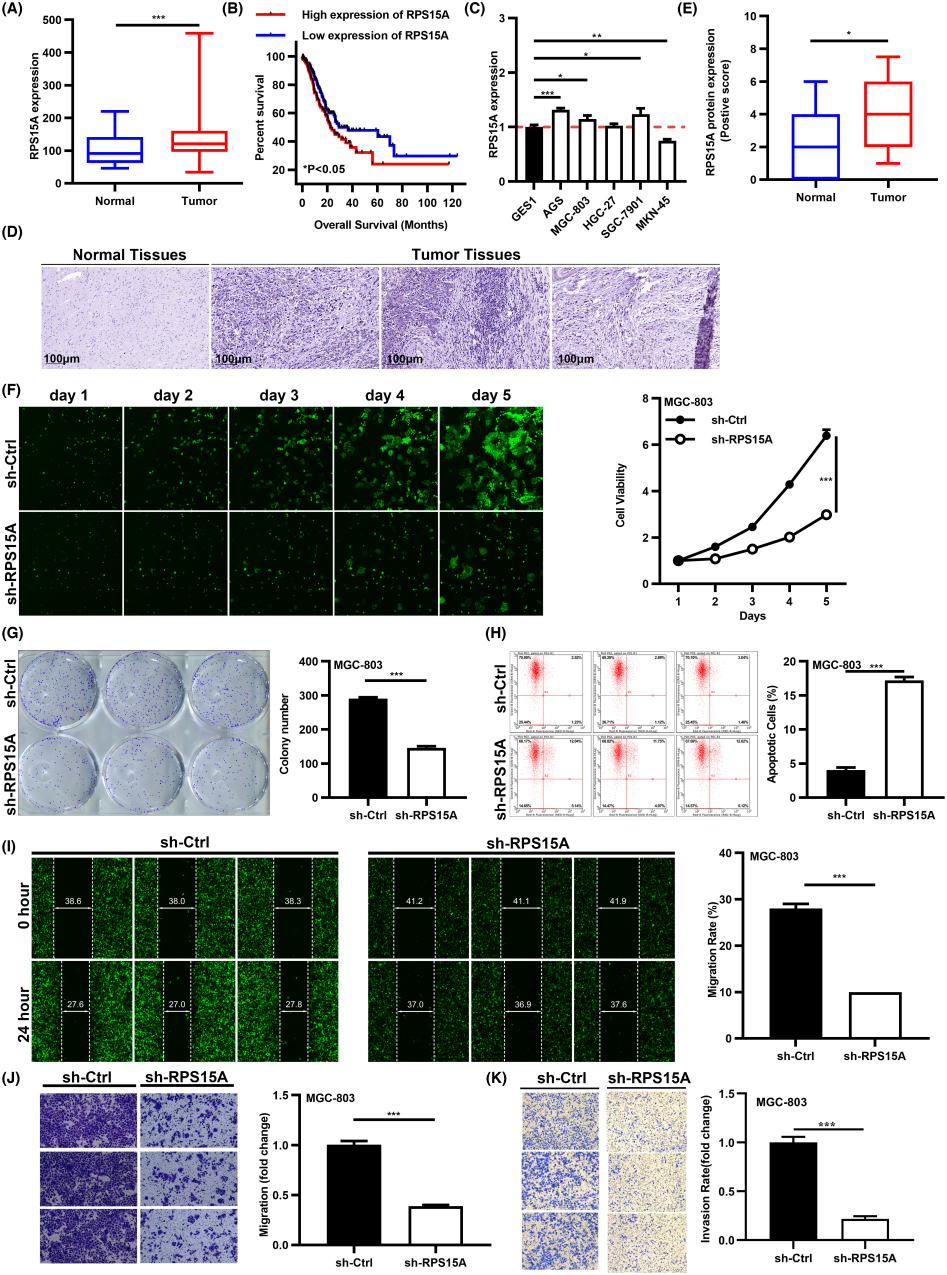
RPS15A knockdown inhibits the phenotypic functions of GC. (A) RPS15A expression in GC and normal tissues from TCGA. (B) Kaplan–Meier plots of GC patients with high and low expression of RPS15A. (C) RPS15A expression in GC and GES‐1 cells. (D, E) IHC staining of RPS15A in GC and normal tissues. Scale bars: 100 μm. (F, G) The proliferation ability of RPS15A was measured by (F) Celigo cell counting assay and (G) Colony formation assay. (H) Annexin V‐APC staining in sh‐RPS15A and sh‐Ctrl groups. (I–K) The migration and invasion ability of MGC‐803 cells was detected by (I) Wound‐healing assay (scale bar: 1 μm) (J, K) and Transwell assay (100×). *p < 0.05, **p < 0.01, and ***p < 0.001.

Then, we tried to find out whether RPS15A contributed to GC progression. RPS15A expression was significantly increased in GC (Figure 4A). High expression of RPS15A was correlated with low OS rate in GC according to Kaplan–Meier survival analysis (Figure 4B). RPS15A expression was increased in most GC cells (Figure 4C). IHC staining confirmed that RPS15A expression in GC was higher than in normal tissues (Figure 4D,E). qRT‐PCR and WB indicated that RPS15A was decreased by shRNA in MGC‐803 cells successfully (Figure S1E,F). Through Celigo cell counting and Colony formation assays, the proliferation ability of GC cells was weakened significantly after RPS15A knockdown (Figure 4F,G). The proportion of apoptotic GC cells in sh‐RPS15A group was higher than in sh‐Ctrl group (Figure 4H). Wound‐healing and Transwell assays proved that migration and invasion of GC cells was suppressed after RPS15A knockdown (Figure 4I–K). Moreover, we overexpressed RPS15A in GC cells (Figure S1G,H). The phenotypic functions of GC cells after RPS15A overexpression were detected (Figure S4A–C). The above results revealed the functions of RPS15A in GC.

### 3.5 Overexpression of RPS15A reverses the changes of MCM8 knockdown in GC cells

**FIGURE 5:**
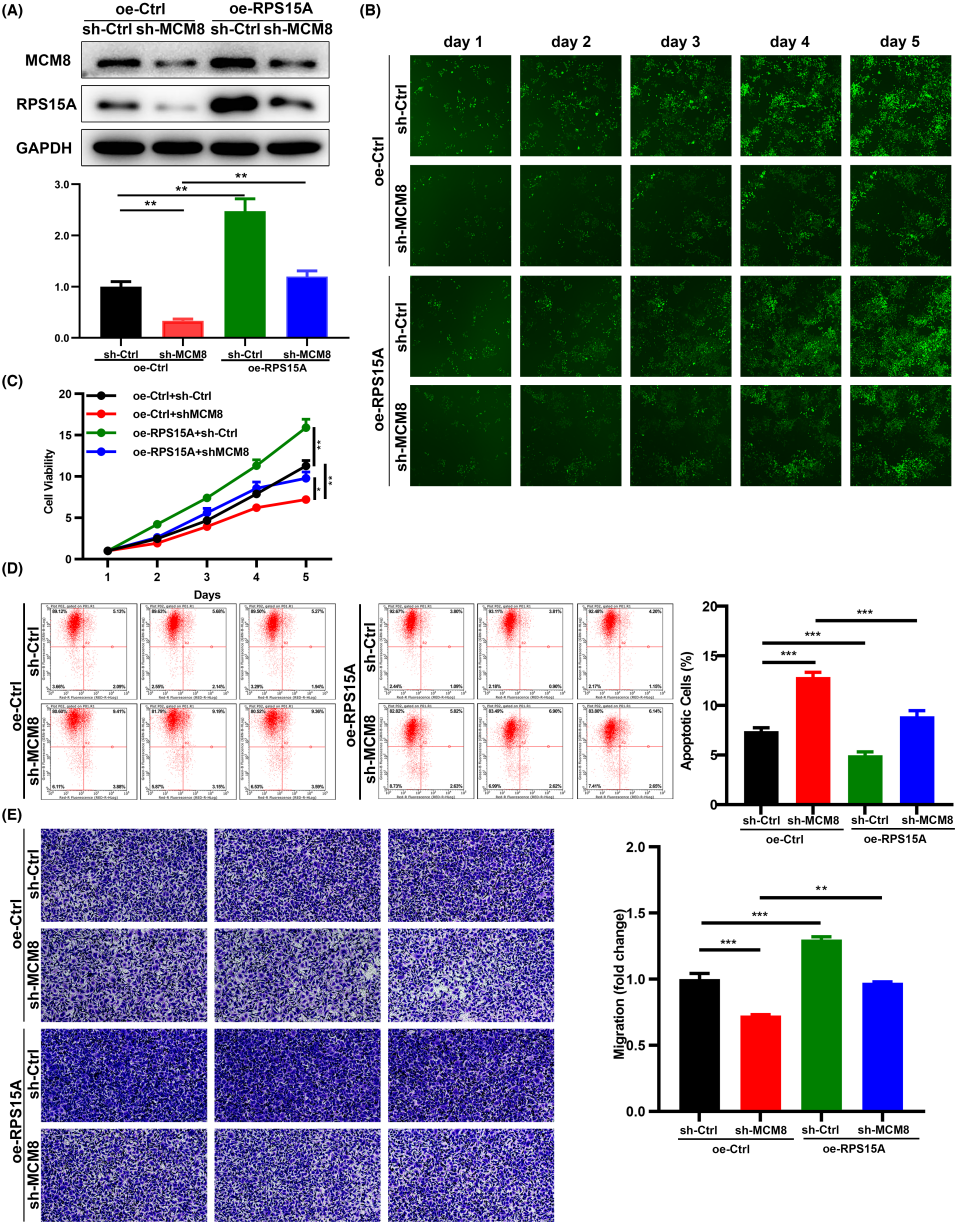
Overexpression of RPS15A reverses the changes of MCM8 knockdown in GC cells. (A) The transfection efficiency was detected by WB. (B–E) sh‐Ctrl+oe‐Ctrl, sh‐Ctrl+oe‐RPS15A, sh‐MCM8+oe‐Ctrl, and sh‐MCM8+oe‐RPS15A groups were established in AGS cells. The loss or gain of function experiments showed that RPS15A reversed the changes of MCM8 knockdown in GC cells, including (B, C) proliferation, (D) apoptosis, and (E) migration (100×). *p < 0.05, **p < 0.01, and ***p < 0.001.

We established sh‐Ctrl+oe‐Ctrl, sh‐Ctrl+oe‐RPS15A, sh‐MCM8+oe‐Ctrl, and sh‐MCM8+oe‐RPS15A groups in AGS cells. The transfection efficiency was detected by WB (Figure 5A). The changes caused by RPS15A overexpression in MCM8 knockdown cells were determined by Celigo cell counting, Annexin V staining, and Transwell assays. These gene gain or loss of function experiments showed that RPS15A reversed the changes of MCM8 knockdown in GC cells (Figure 5B–E). In conclusion, the regulation of MCM8 in GC was realized by RPS15A.

### 3.6 MCM8/RPS15A axis promotes phosphorylation of P38α, LYN, and p70S6K in GC

**FIGURE 6:**
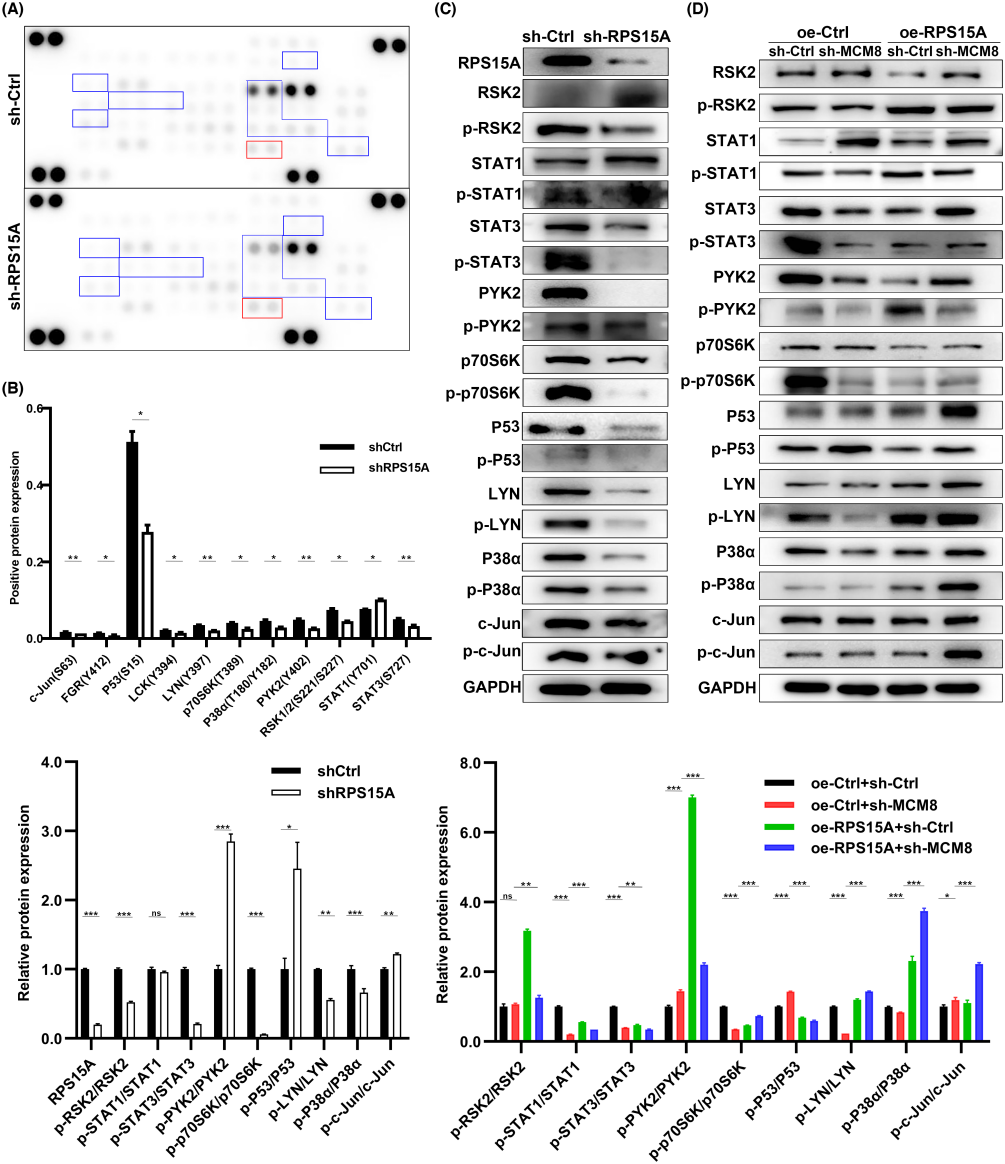
MCM8/RPS15A axis promotes P38α, LYN, and p70S6K phosphorylation in GC. (A, B) Human Phospho‐Kinase Array Kit (ARY003C) was used to detect changes in phosphorylation of key proteins after RPS15A knockdown. (C, D) WB was applied to detect RPS15A, P53, LYN, P38α, c‐Jun, STAT1, STAT3, PYK2, p70S6K and their phosphorylated forms. *p < 0.05, **p < 0.01, and ***p < 0.001.

To further clarify the concrete mechanisms of MCM8 in GC, we used Human Phospho‐Kinase Array Kit (ARY003C) to detect changes in phosphorylation of key proteins after RPS15A knockdown. The kit containing 39 human phosphorylated kinases (Table S1). The phosphorylation of STAT1 (Y701) was upregulated significantly. The phosphorylation of c‐Jun (S63), Fgr (Y412), P53 (S15), Lck (Y394), LYN (Y397), p70S6K (T389), P38α (T180/Y182), PYK2 (Y402), RSK1/2 (S221/S227), and STAT3 (S727) were downregulated (Figure 6A,B). Next, we used WB to investigate the expression of related proteins and their phosphorylated proteins in sh‐Ctrl, sh‐RPS15A, sh‐Ctrl+oe‐Ctrl, sh‐MCM8+oe‐Ctrl, sh‐Ctrl+oe‐RPS15A, and sh‐MCM8+oe‐RPS15A groups. It showed that the expression of phosphorylated P38α (p‐P38α), LYN (p‐LYN), and p70S6K (p‐p70S6K) were decreased in sh‐RPS15A or sh‐MCM8 group. Meanwhile, RPS15A overexpression reversed the reduction of p‐P38α, p‐LYN, and p‐p70S6K induced by MCM8 knockdown (Figure 6C,D). Therefore, we speculated that MCM8/RPS15A axis may promote GC process by facilitating phosphorylation of P38α, LYN, and p70S6K.

### 3.7 Knockdown of MCM8 attenuates GC growth in vivo and the expression of RPS15A, p‐P38α, p‐LYN, and p‐p70S6K

**FIGURE 7:**
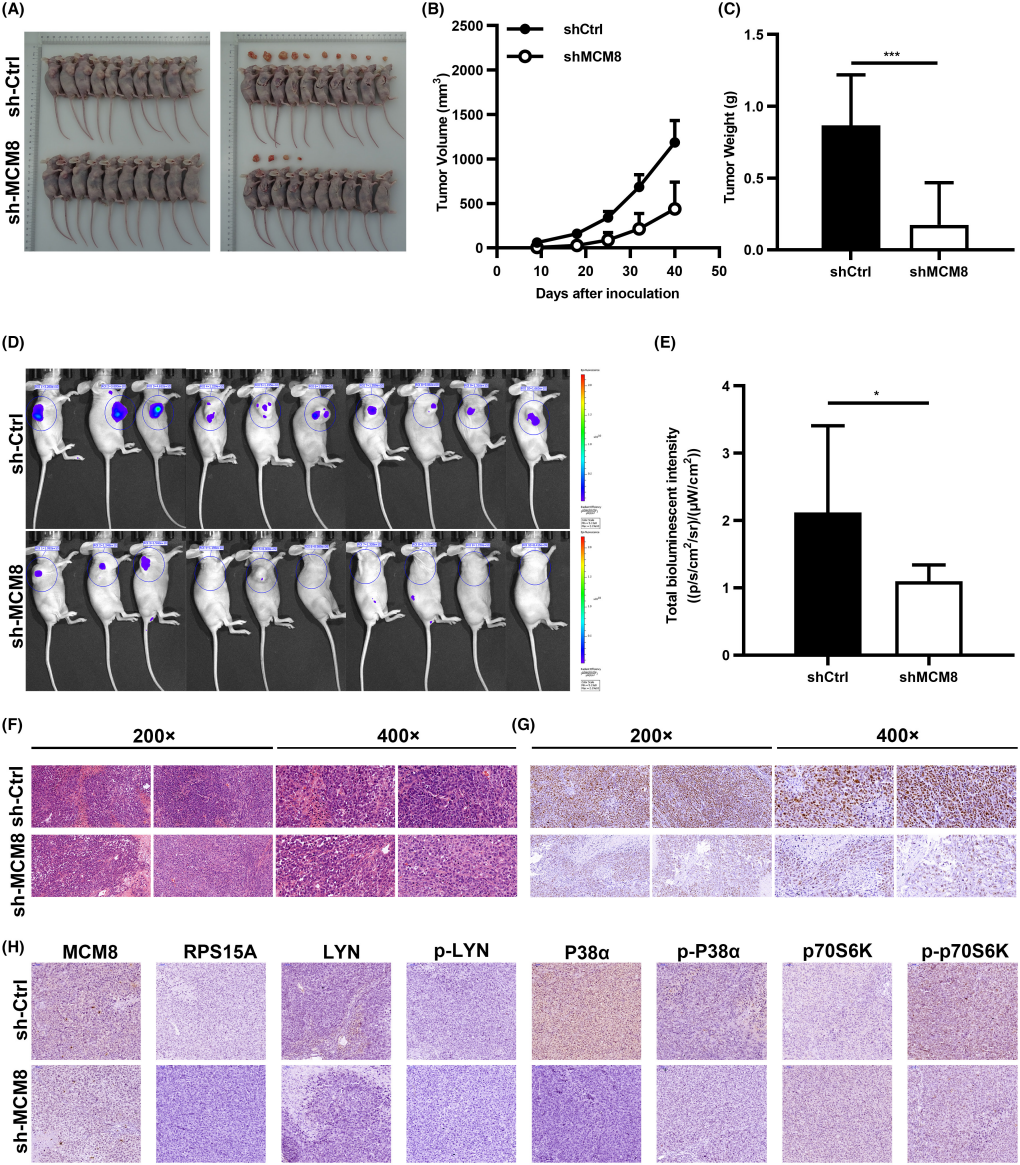
Knockdown of MCM8 attenuates GC growth in vivo. (A) The left picture is the subcutaneous tumor mice. The right is the mice with the tumor removed. (B) Tumor volume and (C) weight in sh‐MCM8 and sh‐Ctrl groups. (D, E) The fluorescence images displayed the tumor burden. (F) Ki67 and (G) HE staining were detected in tissue sections. Scale bars: 100 μm. (H) IHC staining of MCM8, RPS15A, P38α, p‐38α, LYN, p‐LYN, p70S6K, and p‐p70S6K in sh‐MCM8 and sh‐Ctrl tissue sections. Scale bars: 100 μm. *p < 0.05, and ***p < 0.001.

The effect of MCM8 knockdown on GC in vivo was further investigated by xenotransplantation mode. MGC‐803 cells with sh‐MCM8 or sh‐Ctrl were injected subcutaneously into the lateral abdomen of nude mice. The left picture is the subcutaneous tumor mice and the right is the mice with the tumor removed (Figure 7A). Results showed that MCM8 knockdown reduced tumor volume and weight (Figure 7B,C). The fluorescence images displayed that tumor burden and fluorescence intensity were reduced in sh‐MCM8 group (Figure 7D,E). Furthermore, Ki67 and HE staining were detected in mouse tumor sections. Lower Ki67 positive staining were detected in sh‐MCM8 tumor sections. It also proves the proliferative ability of MCM8 on GC (Figure 7F,G). Previous studies have reported that P38α, LYN, and p70S6K are protein kinases belonging to the MAP kinase, Src kinase, and AGC kinase, respectively.^23^, ^24^, ^25^ They regulate cellular processes through activating phosphorylation themselves and promoting phosphorylation of other targets.^25^, ^26^, ^27^ Accordingly, we performed IHC staining on mouse tumor sections to detect P38α, LYN, p70S6K and their phosphorylated proteins. It showed that p‐P38α, p‐LYN, and p‐p70S6K, and RPS15A were decreased after MCM8 knockdown (Figure 7H). Taken together, MCM8 knockdown inhibits GC growth in vivo and the expression of RPS15A, p‐P38α, p‐LYN, and p‐p70S6K.

## 4 DISCUSSION

In this study, MCM8 was expressed highly in GC from TCGA and our data. High MCM8 expression was correlated with poor prognosis. MCM8 knockdown inhibited the proliferation, migration, and invasion while promoting apoptosis of GC cells. In addition, MCM8 knockdown suppressed GC growth in vivo. Mechanistically, RPS15A was the target of MCM8 in GC. The functional experiments of RPS15A confirmed its oncogenic role in GC. We also found that MCM8 protein combined with RPS15A protein in GC. Moreover, MCM8/RPS15A increased the expression of three kinases including p‐P38α, p‐LYN, and p‐p70S6K in GC. Collectively, MCM8 is an oncogene and promotes GC progression through RPS15A.

In recent years, the functions of MCMs in tumor progression have caught researchers' attention.^28^, ^29^, ^30^ MCM2 and MCM4 suppress CRC and HPV‐type of cervical cancer progression, respectively.^28^, ^29^, ^30^ MCM8 is involved in the initiation and extension of DNA replication. It can constitute an iso‐hexameric ring complex with MCM9.^31^ It promotes the HR through promoting RAD51 recombinase recruitment at DNA damage sites and splicing DNA double‐strand breaks. This function can prevent genomic instability and cancer susceptibility caused by double‐strand breaks accumulation.^32^, ^33^, ^34^, ^35^

To date, there have been few reports of MCM8 in digestive system cancers. MCM8 mutations often promote benign disease progression. For example, MCM8 mutation leads to defects in chromosome breakage and repair of fibroblasts in the premature ovarian failure.^35^ Previous studies have only confirmed that MCM8 promotes the phenotypic function of GC cells.^22^ The mechanisms of MCM8 in GC are still unknown. We explore the mechanisms of MCM8 in GC for the first time, and regards RPS15A as the target of MCM8 promoting GC progression. RPS15A is a highly conserved protein belonging to the 40S ribosomal subunit. It is essential for ribosome assembly and translation.^36^ Additionally, RPS15A is indispensable for cell survival and proliferation. It promotes cap mRNA binding with 40S ribosomal subunit during early translation by interacting with the cap binding subunit of eukaryotic initiation factor 4F.^37^, ^38^ Previous studies have reported that RPS15A is a carcinogenic among digestive system tumors.^39^, ^40^, ^41^ For example, RPS15A promotes HCC development,^39^, ^40^ and RPS15A promotes the malignant progression of CRC through the P53 signaling pathway.^41^ Our subsequent functional experiments confirm the oncogenic role of RPS15A in GC and the reversal effect of RPS15A on MCM8 knockdown. In conclusion, MCM8 promotes GC progress through RPS15A. In addition, KEGG pathway enrichment analysis indicates that MCM8/RPS15A axis also plays an important role in cell senescence and MicroRNA in cancer (Figure S2C). Therefore, MCM8/RPS15A axis may promote GC progression in different ways.

Previous studies have proved that MCM8 plays roles in the nucleus by binding with other proteins. For example, MCM8 protein binds with cyclin D1 protein in the nucleus to promote cell cycle.^42^ Therefore, MCM8 and RPS15A might have a binding relationship at the protein level. In this study, Co‐IP assay was performed to verify this hypothesis. It has been reported that MCM8 protein colocalizes on a specific DNA segment of the c‐MYC replication initiation zone with Cdc6.^43^ It suggests that the main functional site of MCM8 protein may be in the nucleus. Nevertheless, we do not know the specific intracellular localization where MCM8 protein binds with RPS15A protein in GC cells. It still needs to be further explored through more experiments such as multiplex immunofluorescence assays.

In this study, the phosphorylation level of P38α, LYN, and p70S6K was elevated in GC. It has been published that unphosphorylated LYN reduces p70S6K expression by inhibiting AKT/mTOR phosphorylation, which ultimately leads to impede GC progression.^44^ Further more, LYN and p70S6K play oncogenic role through phosphorylation.^44^ The phosphorylation activation of p70S6K was inhibited by deleted in liver cancer 2 (DLC2) gene through Rho GTPase‐activating protein, resulting in the suppression of HCC cell growth.^45^ The phosphorylation of LYN kinases was activated by high extracellular matrix stiffness to promote breast tumor invasion and metastasis.^46^ In this study, the expression of p‐P38α, p‐LYN, and p‐p70S6K were increased in GC. We speculated that p‐LYN may promote p70S6K phosphorylation by increasing AKT/mTOR phosphorylation levels in GC. A recent study showed that p‐P38α/p‐AKT promotes the growth and metastasis of CRC.^47^, ^48^ However, the functions of p‐P38α/p‐AKT in GC are unknown. Whether it is involved in the axis we have suggested remains to be confirmed.

Furthermore, p70S6K, also called RPS6KB1, can phosphorylate and activate RPS6 to promote cell growth.^49^, ^50^ Both RPS15A and RPS6 are components of the 40S subunit of the ribosome.^51^, ^52^, ^53^ Activated RPS6 and RPS15A promote ribosome function in GC cell. In esophageal squamous cell carcinoma, dihydroartemisinin weakens p‐mTOR, p‐p70S6K, and p‐RPS6 to inhibit tumor growth.^54^ In GC, the correlation between p‐p70S6K and ribosome function of RPS15A still needs to be further explored. The upstream mechanisms of MCM8 in cancer are studied poorly. We need additional studies and larger numbers of GC samples to further investigate. MCM8 has the potential to be one of the targets for GC treatment. However, MCM8 targeting therapy and medicinal development in GC patients still needs further research. In conclusion, MCM8 was significantly upregulated and predicted poor prognosis in GC. MCM8 knockdown inhibited proliferation and migration while promoting apoptosis. MCM8 promoted GC progression through RPS15A.

## AUTHOR CONTRIBUTIONS

**Lixian Ding:** Conceptualization (lead); data curation (lead); formal analysis (lead); validation (lead); writing – original draft (lead). **Mingjun Sun:** Data curation (equal); formal analysis (equal). **Yanyan Sun:** Data curation (equal); formal analysis (equal); writing – original draft (equal). **Jinxing Li:** Data curation (equal). **Zhicheng Zhang:** Formal analysis (equal); writing – original draft (equal). **Shuwei Dang:** Supervision (equal); validation (equal). **Jinning Zhang:** Writing – original draft (supporting). **Bang Yang:** Data curation (supporting). **Youlin Dai:** Data curation (equal). **Qinghao Zhou:** Data curation (supporting). **Dazhi Zhou:** Writing – original draft (supporting). **Encheng Li:** Writing – original draft (supporting). **Shuqi Peng:** Data curation (supporting). **Guodong Li:** Supervision (lead); writing – review and editing (lead).

## FUNDING INFORMATION

This research was funded by National Natural Science Foundation of China (82072673) and Natural Science Foundation of Heilongjiang Province (LH2022H032).

## CONFLICT OF INTEREST STATEMENT

The authors declare no conflicts of interest.

### ETHICS STATEMENT

This study was approved by the Ethics Committee of The Fourth Affiliated Hospital of Harbin Medical University (YXLLSC‐2018‐01). Informed consent was obtained from all patients.

## Supporting information


Figure S1.

Table S1.

## Data Availability

The data generated in this study are available upon request from the corresponding author.
