# Supplementary material for: MCM8 promotes gastric cancer progression through RPS15A and predicts poor prognosis
Source: Cancer Med. 2024 Jul 10;13(13):e7424. doi: 10.1002/cam4.7424 (PMC11236911; doi:10.1002/cam4.7424)
Supplement: Supplementary file 1 — Figure S1. [file CAM4-13-e7424-s002.pdf]

Figure S1

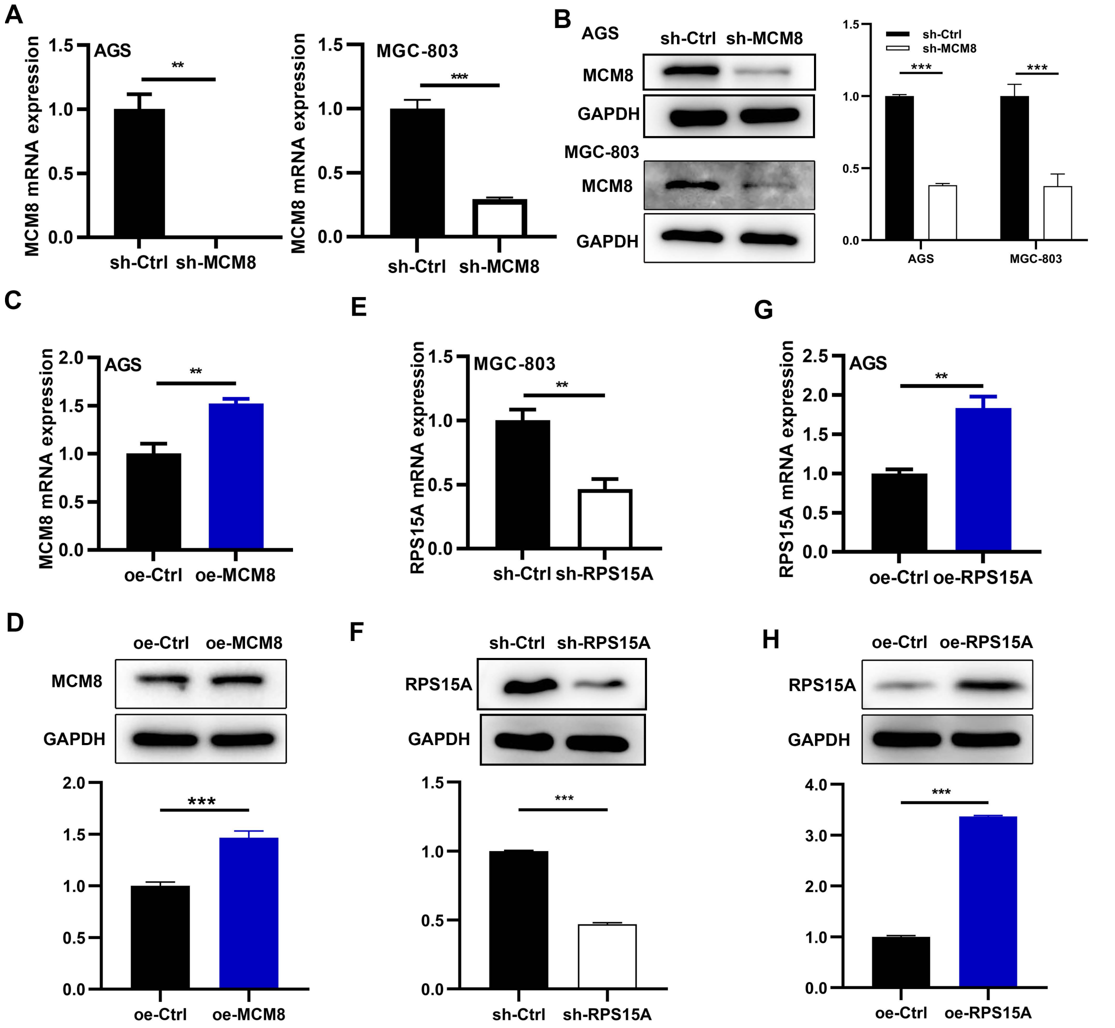

Figure S1. Validation of transfection efficiency. **(A-B)** **(A)** qRT-PCR and **(B)** WB were used to determine the knockdown efficiency of MCM8 in AGS and MGC-803 cells. **(C-D)** MCM8 expression was detected by **(C)** qRT-PCR and **(D)** WB in AGS cells. **(E-F)** RPS15A knockdown efficiency was verified by **(E)** qRT-PCR and **(F)** WB in MGC-803 cells. **(G-H)** RPS15A overexpression efficiency was measured by **(G)** qRT-PCR and **(H)** WB in AGS cells. $^{**}P < 0.01$ and $^{***}P < 0.001$.

Figure S2

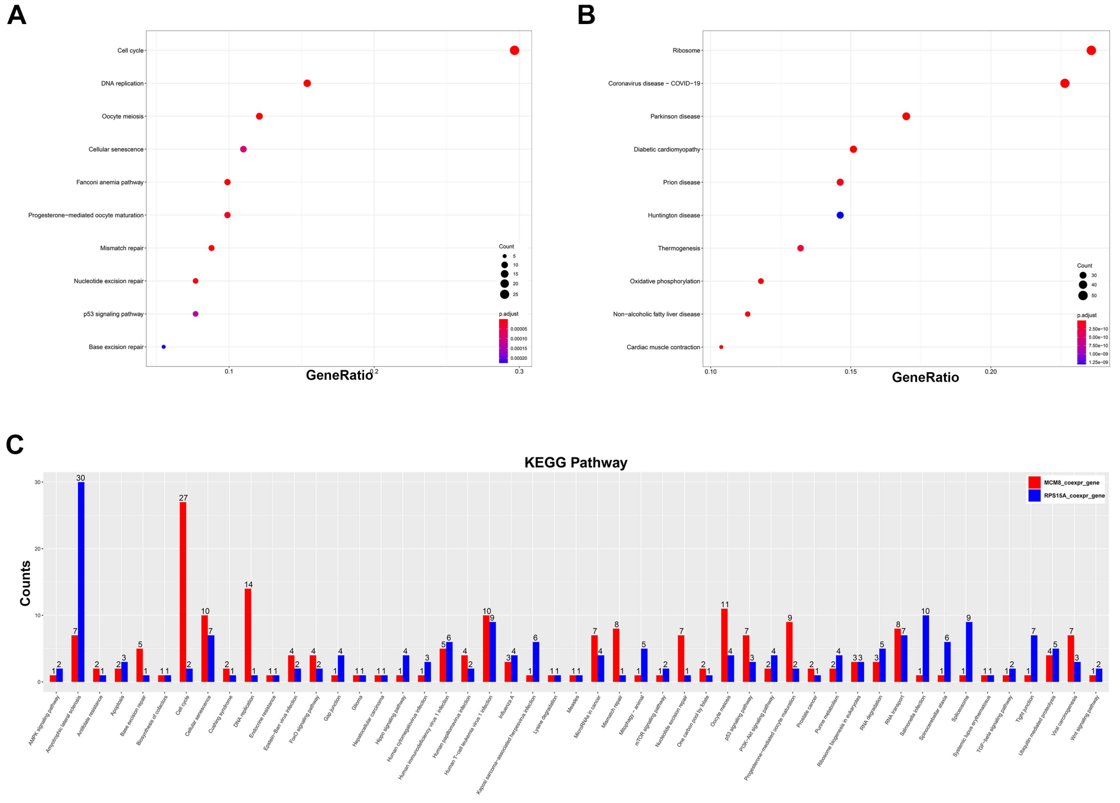

Figure S2. KEGG pathway enrichment analysis. **(A-C)** KEGG pathway enrichment analysis revealed the pathway enrichment of **(A)** MCM8 co-expression genes, **(B)** RPS15A co-expression genes, **(C)** MCM8 and RPS15A co-expression genes.

Figure S3

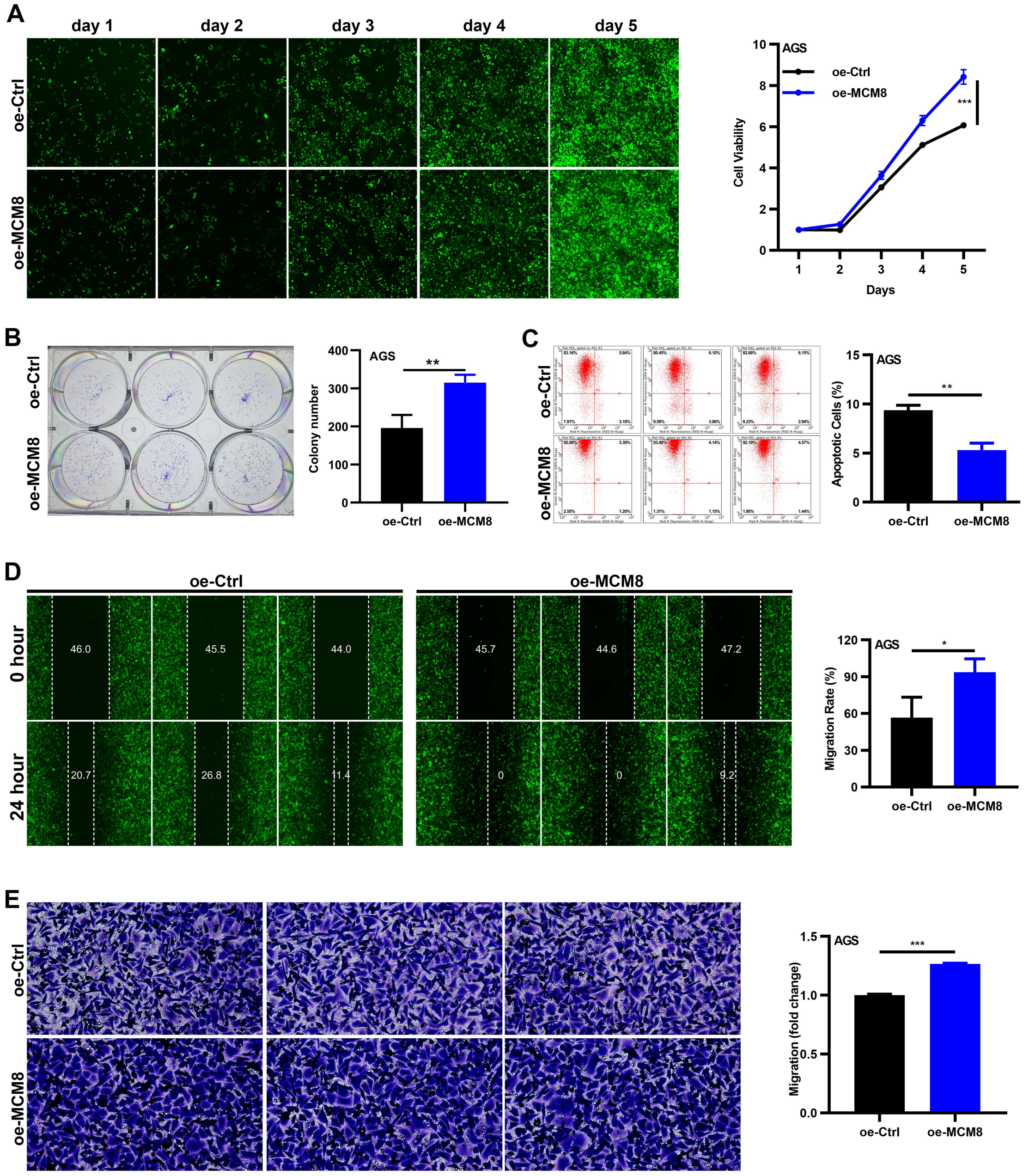

Figure S3. MCM8 overexpression promotes the phenotypic functions of GC. (A-B) MCM8 overexpression enhanced the proliferation of AGS cells through (A) Celigo cell counting assay and (B) Colony formation assay. (C) Flow cytometry results showed that MCM8 overexpression inhibited the apoptosis ability of AGS cells. (D-E) (D) Wound healing assay (Scale bar: 1 μm) and (E) Transwell assay (200×) were used for migration evaluation of AGS cells. *P < 0.05, **P < 0.01, and ***P < 0.001.

Figure S4

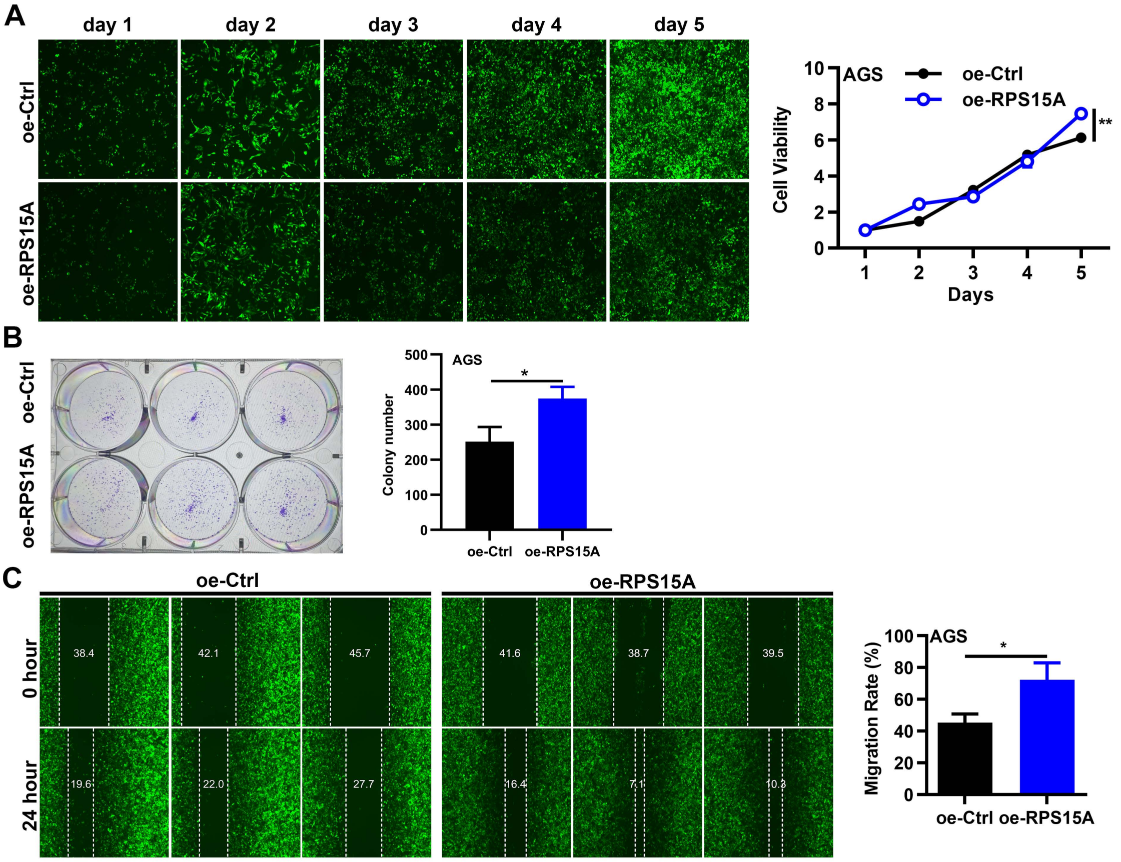

Figure S4. RPS15A overexpression facilitates the phenotypic functions of GC. **(A-B)** **(A)** Celigo cell counting assay and **(B)** Colony formation assay were performed to detect the proliferation ability of AGS cells. **(C)** The migration ability of RPS15A was measured by Transwell assay. *P < 0.05 and **P < 0.01.
