# Supplementary material for: MCM8 promotes gastric cancer progression through RPS15A and predicts poor prognosis
Source: Cancer Med. 2024 Jul 10;13(13):e7424. doi: 10.1002/cam4.7424 (PMC11236911; doi:10.1002/cam4.7424)
Supplement: Supplementary file 2 — Table S1. [file CAM4-13-e7424-s001.docx]

Table S1. The 11 positive phosphorylated kinase in the Human Phospho-Kinase Array Kit (ARY003C) were labeled.

|  | **A** | **B** | **C** | **D** | **E** | **F** | **G** |
| --- | --- | --- | --- | --- | --- | --- | --- |
| **1** | Refer Sp |  |  |  |  |  | Refer Sp |
| **2** | Refer Sp |  |  |  |  |  | Refer Sp |
| **3** |  | CREB (S133) | Fgr(Y412) | JNK1/2/3 (T183/Y185  , T221/Y223) | p38α  (T180/Y182) | STAT2(Y689) | β-Catenin |
| **4** |  | CREB (S133) | Fgr(Y412) | JNK1/2/3 (T183/Y185  , T221/Y223) | p38α  (T180/Y182) | STAT2(Y689) | β-Catenin |
| **5** |  | EGFR (Y1086) | GSK-3α/β  (S21/S9) | Lck(Y394) | PDGF Rβ  (Y751) | STAT5a/b  (Y694/Y699) |  |
| **6** |  | EGFR (Y1086) | GSK-3α/β  (S21/S9) | Lck(Y394) | PDGF Rβ  (Y751) | STAT5a/b  (Y694/Y699) |  |
| **7** |  | eNOS (S1177) | GSK-3β  (S9) | Lyn(Y397) | PLC-γ1  (Y783) | WNK1(T60) |  |
| **8** |  | eNOS (S1177) | GSK-3β  (S9) | Lyn(Y397) | PLC-γ1  (Y783) | WNK1(T60) |  |
| **9** |  | ERK1/2  (T202/Y204, T185/Y187) | Hsp27 (S78/S82) | Msk1/2  (S376/S360  ) | Src(Y419) | Yes(Y426) | PBS  (Negative Control) |
| **10** |  | ERK1/2  (T202/Y204, T185/Y187) | Hsp27 (S78/S82) | Msk1/2  (S376/S360  ) | Src(Y419) | Yes(Y426) | PBS  (Negative Control) |

| **11** | Akt1/2/3  (T308) | Chk-2  (T68) | p53(S15) | p70 S6K  (T389) | PYK2  (Y402) | STAT1  (Y701) | STAT6  (Y641) |
| --- | --- | --- | --- | --- | --- | --- | --- |
| **12** | Akt1/2/3  (T308) | Chk-2  (T68) | p53(S15) | p70 S6K (T389) | PYK2 (Y402) | STAT1 (Y701) | STAT6 (Y641) |
| **13** | Akt1/2/3  (S473) | c-jun(S63) | p53(S46) | p70 S6K (T421/S424  ) | RSK1/2 (S221/S227) | STAT3 (Y705) | Hsp60 |
| **14** | Akt1/2/3  (S473) | c-jun(S63) | p53(S46) | p70 S6K (T421/S424  ) | RSK1/2 (S221/S227) | STAT3 (Y705) | Hsp60 |
| **15** |  |  | p53(S392) | PRAS40 (T246) | RSK1/2/3 (S380/S386/ S377) | STAT3 (S727) |  |
| **16** |  |  | p53(S392) | PRAS40 (T246) | RSK1/2/3 (S380/S386/ S377) | STAT3 (S727) |  |
| **17** | Refer Sp |  |  |  |  |  | PBS  (Negative Control) |
| **18** | Refer Sp |  |  |  |  |  | PBS  (Negative Control) |
